# Mobile Health Requirements for the Occupational Health Assessment of Health Care Professionals: Delphi Study

**DOI:** 10.2196/40327

**Published:** 2023-05-31

**Authors:** Ana Belen Naranjo-Saucedo, German Antonio Escobar-Rodriguez, Carmen Tabernero, Esther Cuadrado, Carlos Luis Parra-Calderon, Alicia Arenas

**Affiliations:** 1 Institute of Biomedicine of Seville/Virgen del Rocío University Hospital Consejo Superior de Investigaciones Científicas/University of Seville Sevilla Spain; 2 Maimonides Biomedical Research Institute of Córdoba Córdoba Spain; 3 Department of Social Psychology and Anthropology Instituto de Neurociencias de Castilla y León Campus of the University of Salamanca-Miguel de Unamuno Salamanca Spain; 4 Faculty of Educational Sciences and Psychology University of Córdoba Córdoba Spain; 5 Department of Social Psychology University of Seville Seville Spain

**Keywords:** occupational health assessment, psychosocial risks, health care professionals, mobile health, mHealth, Delphi method

## Abstract

**Background:**

In recent years, owing to the COVID-19 pandemic, awareness of the high level of stress among health care professionals has increased, and research in this area has intensified. Hospital staff members have historically been known to work in an environment involving high emotional demands, time pressure, and workload. Furthermore, the pandemic has increased the strain experienced by health care professionals owing to the high number of people they need to manage and, on many occasions, the limited available resources with which they must carry out their functions. These psychosocial risks are not always well dealt with by the organization or the professionals themselves. Therefore, it is necessary to have tools to assess these psychosocial risks and to optimize the management of this demand from health care professionals. Digital health, and more specifically, mobile health (mHealth), is presented as a health care modality that can contribute greatly to respond to these unmet needs.

**Objective:**

We aimed to analyze whether mHealth tools can provide value for the study and management of psychosocial risks in health care professionals, and assess the requirements of these tools.

**Methods:**

A Delphi study was carried out to determine the opinions of experts on the relevance of using mHealth tools to evaluate physiological indicators and psychosocial factors in order to assess occupational health, and specifically, stress and burnout, in health care professionals. The study included 58 experts with knowledge and experience in occupational risk prevention, psychosocial work, and health-related technology, as well as health professionals from private and public sectors.

**Results:**

Our data suggested that there is still controversy about the roles that organizations play in occupational risk prevention in general and psychosocial risks in particular. An adequate assessment of the stress levels and psychosocial factors can help improve employees’ well-being. Moreover, making occupational health evaluations available to the team would positively affect employees by increasing their feelings of being taken into account by the organization. This assessment can be improved with mHealth tools that identify and quickly highlight the difficulties or problems that occur among staff and work teams. However, to achieve good adherence and participation in occupational health and safety evaluations, experts consider that it is essential to ensure the privacy of professionals and to develop feelings of being supported by their supervisors.

**Conclusions:**

For years, mHealth has been used mainly to propose intervention programs to improve occupational health. Our research highlights the usefulness of these tools for evaluating psychosocial risks in a preliminary and essential phase of approaches to improve the health and well-being of professionals in health care settings. The most urgent requirements these tools must meet are those aimed at protecting the confidentiality and privacy of measurements.

## Introduction

It is undisputed that health care professionals often experience high levels of work-related stress. They have a diversity of work schedules, high workloads, night shifts that lead to sleep deprivation, work-life imbalance, feelings of isolation, and low control over the content of tasks or decisions related to their work [1]. All of these require the ability to control and manage physiological and psychological stress, and they experience disturbances in their self-regulation capacities and sometimes fail to fulfill their duties and responsibilities [2]. Job stress causes the appearance of health problems in these professionals, leading to changes in their place of work and even creating the need for these professionals to leave their profession. This situation has worsened in recent years. The emotional well-being of health care professionals is critical. It is even more relevant today given the effects that the COVID-19 pandemic has had on health care management and patient care, especially in the work environment, due to both the psychological impact on professionals themselves and the possible consequences regarding job performance and patient care [3]. Studies have found that hospital personnel were significantly affected during the pandemic and reported high levels of concern given the fear of infection and the possible consequences of the disease on their physical and mental health [4,5]. These concerns included fear, insecurity, anxiety, and compassion fatigue, with the latter caused by the cost of worrying about others or their emotional pain associated with the aim of alleviating the suffering of patients [4]. On the other hand, workplace issues can play a crucial role in modifying or worsening the mental health of people who face this pandemic scenario. Among them, job insecurity, periods of isolation, and uncertainty are factors in the workplace that most affect health care workers [5].

To carry out appropriate interventions aimed at reducing work-related stress, the professional situation must first be adequately assessed and diagnosed. However, work overload is a barrier to performing work stress measurements, so it is essential to find a way to achieve such measurements with minimal harm to professionals and their work performance. The key may lie in technology. Currently, there are more research initiatives and initiatives involving the development of new technological tools in this specific population to assess the stress experienced by these professionals [6]. In this sense, we found some studies in which digital tools were used as applications to assess and improve the well-being of professionals [7,8]. These tools are often designed to offer training strategies to improve stress management at work. However, to our knowledge, tools focused on diagnosing the health of workers are scarce.

In the scientific literature, there are studies focused on mobile health (mHealth) tools to assess the work environment and its psychosocial aspects, as well as how stress affects the health of professionals. The results of current research on the organizational health of hospital staff revealed high levels of stress [9] and burnout [10], as well as other psychological problems, such as anxiety and depression [11], leading to a high turnover of employees and a high number of sick leaves [12], and there have even been occasions where the treatment of patients or their family members has been considerably reduced [13]. Furthermore, the situation since the COVID-19 pandemic has further aggravated this reality [14]. Some tools offer individualized intervention programs for professionals [15,16]. There is limited literature on tools that perform occupational health measurements in professionals, and mHealth tools, such as mobile apps, often focus on the implementation of mindfulness intervention programs [17], breath control programs, or meditation intervention programs [18]. It should be noted that the use of wearable technology allows the recording of physiological variables in real time with great success [19]. These are small devices that capture biometric data from users over a prolonged period of time in natural environments and record variables [20], such as heart rate, respiratory rate, and hours of sleep, allowing the assessment of the stress levels and well-being of workers [21]. In contrast, mHealth tools focused on intervention and program implementation highlight approaches focused on mindfulness training [10], breathing control [22], and learning techniques to cope with stress and resilience [23] in difficult situations that arise on the job and in the lives of workers. Learning these techniques can help reduce stress levels among trainees and improve their well-being [24].

We were interested in exploring how mHealth tools should be focused on the identification and evaluation of psychosocial factors, such as stress and burnout, in an essential preliminary phase for interventions. We posed the following research question to experts: Do mHealth tools applied to occupational health allow you to understand and manage psychosocial risks among health care professionals in their organizations? Our study explored their opinions about the use of these tools in organizations to assess the occupational health of professionals and determined the characteristics that these tools must have to be accepted by workers.

## Methods

### Delphi Process

The Delphi method was applied in a group setting to obtain consensus on the opinions of a group of experts through a series of intensive questionnaires with controlled opinion feedback [25]. The approach was applied to obtain expert opinion on the application of mHealth tools. It is commonly used in the fields of technological and social forecasting, social diagnosis, consensual interpretation of social or health realities, communication, and participation [26]. We believe that the Delphi methodology is suitable for answering our research question because of its ability to obtain expert consensus, its application in technological and social fields, and its ability to obtain accurate and reliable data. The response options in the Delphi method usually follow a 5-choice Likert scale [27].

**Figure 1:**
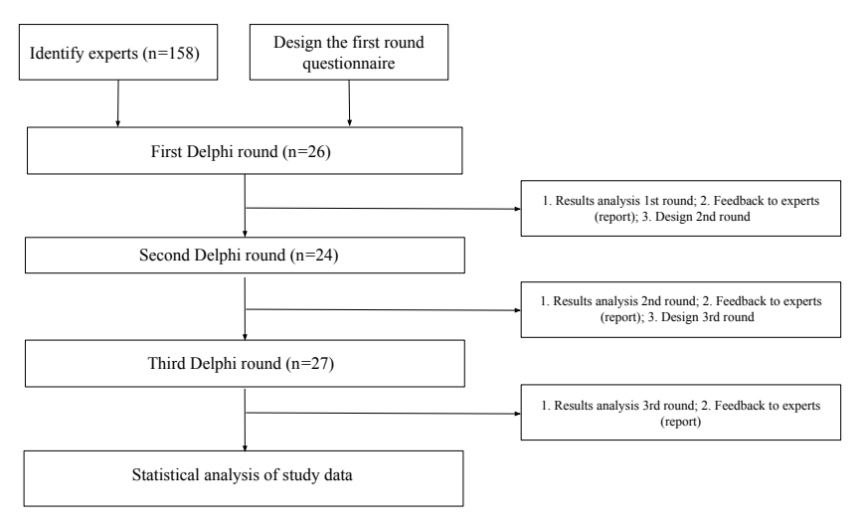
Delphi process in this study.

Several steps were taken to carry out this study, as shown in Figure 1.

### Design of the Expert Panel

The first phase of the Delphi method consisted of identifying experts to create a coherent panel of experts according to the subject matter of the study. After a bibliographic review, a team of approximately 60 experts with knowledge and experience in occupational risk prevention, psychosocial work, and health-related technology, as well as health professionals was selected. For this purpose, professionals belonging to the academic fields of social psychology, computer engineering, and industrial engineering were identified. Health care and nonhealth care professionals were selected. Finally, experts from the private business sector were included. Different levels were considered, including company managers, middle managers, and operating core team members. In the case of experts from the labor or academic field, experience in the use of new technologies applied to health, the field of occupational risk prevention, or the psychosocial area was required. The characteristics of the experts who finally participated are detailed in Table 1.

An average of 9 academic experts, 9 health experts, and 7.66 experts from the business sector participated in each round. In our study, 85% (23/27) of the respondents had more than 10 years of experience.

**Figure 2:**
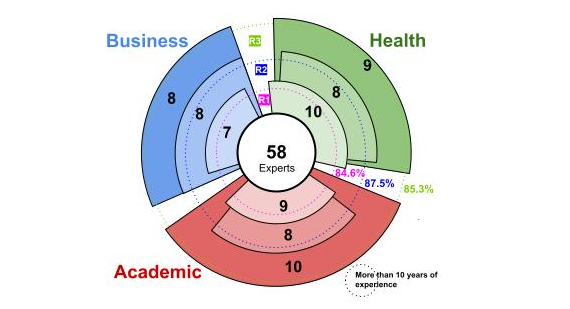
Graphical representation of expert participation.

In round 1, 26 experts participated (45% response rate), of whom 9 were from the academic field, 10 from the public health field, and 7 from the business field related to the health area, and 85% (22/26) had more than 10 years of experience. In round 2, 24 experts participated (41% response rate), with 8 from each area of participation (academic field, public health field, and business field), and 88% (21/24) had more than 10 years of experience. Finally, in round 3, 27 experts participated (46.5% response rate), of whom 10 were from the academic field, 9 from the health field, and 8 from the business field, and 85% (23/27) had more than 10 years of experience. These data are shown graphically in Figure 2.

**Table 1:** Sociodemographic characteristics of the panel of experts.

Variable				Respondents in the first round (n=26), n (%)		Respondents in the second round (n=24), n (%)		Respondents in the third round (n=27), n (%)
**Sector**								
	**Academic**		9 (35)		8 (33)		10 (37)	
		Psychology	3 (33)		3 (38)		3 (30)	
		Industrial engineering	1 (11)		1 (13)		2 (20)	
		Computer engineering	4 (44)		3 (38)		5 (50)	
		Occupational risk prevention	1 (11)		1 (13)		0 (0)	
	**Health care**		10 (39)		8 (33)		9 (33)	
		Health care activities	5 (50)		6 (75)		2 (22)	
		Nonhealth care activities	5 (50)		2 (25)		7 (78)	
		Company managers	5 (50)		4 (50)		5 (56)	
		Middle managers	4 (40)		3 (38)		3 (33)	
		Operating core team members	1 (10)		1 (13)		1 (11)	
	**Business**		7 (27)		8 (33)		8 (30)	
		Company managers	3 (43)		6 (75)		4 (50)	
		Middle managers	4 (57)		2 (25)		4 (50)	
		Operating core team members	0 (0)		0 (0)		0 (0)	
**Experience in** **the sector**								
	1-3 years		1 (4)		2 (8)		2 (7)	
	3-5 years		1 (4)		0 (0)		1 (4)	
	5-10 years		2 (8)		1 (4)		1 (4)	
	>10 years		22 (85)		21 (88)		23 (85)	

### Design of the Questionnaire

A questionnaire was designed to include questions involving the most relevant categories or areas according to the findings obtained in our literature search. These categories were labeled as follows: “occupational health,” “procedure (applicability),” “security and privacy,” and “how should mHealth devices be.”

To ensure that all relevant aspects had been included, the initial questionnaire included 4 open-ended questions to obtain the experts’ opinions on general aspects. The objective was to check whether the information was included in the closed-ended question items or whether, in contrast, it would be convenient to add new categories or issues in the next round. After analyzing the open-ended questions, the category “adherence” was added.

The items presented to the experts were designed according to an ordinal Likert scale with scores ranging from 1 to 5 (1=“strongly disagree” and 5=“strongly agree”). For facilitating the analysis of the results, the ratings were divided into the following 3 levels: (1) disagree (“strongly disagree” and “disagree” responses); (2) neither agree nor disagree (“neither agree nor disagree” responses); and (3) agree (“strongly agree” and “agree” responses) [28].

The questionnaires were developed using computer tools to facilitate the publication and collection of opinions. At the beginning of the surveys, consent was requested to participate. The instructions provided technical terms and their definitions to ensure that participants had the same conceptualizations (detailed in Multimedia Appendix 1). The items of each round were then included, and some questions about the sociodemographic profile of the experts (professional field, years of experience in the sector, and professional group) were provided.

### Description and Procedure of Delphi Rounds

Once the opinions were collected, we analyzed the consensus for each item. Although there is no unanimous definition of consensus, one of the most commonly used is the percentage of agreement. Therefore, some authors, such as Humphrey-Murto et al [29], state that if 70% of experts agree on a response, the item has reasonably reached an agreement of experts to be eliminated for subsequent rounds. To achieve a more robust consensus, it was decided to consider that items with 75% agreement had reached consensus and therefore would not be presented in successive rounds. According to the literature consulted, it was decided to carry out a maximum of 3 iterative rounds, since this is the most frequent. It is estimated that if agreement has not been reached in these 3 rounds, it will not be reached later.

#### Round 1

The objective, methodology, and theoretical background of the study were presented to the experts in the first contact. Similarly, the link to access the first round of the Delphi survey was included.

The first round was launched on June 22, 2021. It consisted of 40 questions. These questions dealt with the following categories: “occupational health,” “procedure (applicability),” “security and privacy,” and “how should mHealth devices be.” Each category consisted of 10 items. Only in this initial round were 4 open-ended questions also included. They were taken into account and incorporated in the next round in case the aspects revealed by experts were not taken into account in the design of the original survey. These questions were as follows: (1) In what ways do you think mHealth systems can be used to assess occupational health? (2) What difficulties can be encountered when applying an occupational health program using mHealth in the health care setting? (3) How do you think the adherence of health care personnel to mHealth tools and interventions could be increased? and (4) What do you think these tools add to the conventionally used tools to assess occupational health in organizations?

#### Round 2

Round 2 was launched on August 10, 2021. A report of the responses provided by the experts in the previous round was sent and included a link to the round 2 survey. It consisted of 15 items whose categories were the same as in the previous round. Based on the responses collected in the open-ended questions of round 1, some new items were added to existing categories in this round, and a new category called “adherence” was incorporated.

#### Round 3

Round 3 was launched on October 5, 2021, and was accompanied by a detailed report of the responses from the previous round. The questionnaire consisted of 5 items belonging to the categories “occupational health” and “security and privacy,” which were the only categories yet to reach consensus.

### Ethical Considerations

This study is part of the research projects “mPRL: mHealth tool for prevention in the area of psychosociology” (reference: PII2019SC0009) and “SALPRO, Analysis of physiological indicators and psychosocial factors in occupational health of health professionals.” Both projects have been approved by the Ethics Committee of the local government (Research Ethics Committee of Cádiz on March 28, 2019, and Research Ethics Committee of the Vírgen Macarena-Virgen del Rocío University Hospitals on December 3, 2019, respectively), and the study complies with the principles set out in the Declaration of Helsinki [30]. Participation was completely anonymous and confidential to guarantee the protection of the privacy of participants and was voluntary. Participants were informed of the aims of the research before they provided their consent to participate. No compensation was provided to study participants.

## Results

### Analysis of the Results of Each Round

#### Round 1

In round 1, agreement was reached on 30 of the 40 items. By analyzing the different sections, the category “occupational health” reached consensus on 5 of the 10 items. Full consensus was reached for the category “procedure (applicability),” so it was not presented again in subsequent rounds. In the category “how should mHealth devices be,” consensus was reached on 11 of the 12 items. In the category “security and privacy,” 6 of the 10 items achieved consensus in this initial round.

The analysis of open-ended questions was carried out by grouping the main themes of each response. Some responses covered aspects of the sections presented in the initial design, and corroborated that the initial design was adequate in those aspects. For example, some experts pointed out expressions, such as “They could be useful instruments for monitoring indicators related to health and stress in real time (P8), and encouraging or stimulating the adoption of healthy habits during working hours,” so we considered it in “occupational health” item OH4. Moreover, the response “one of the cross-cutting difficulties that can be encountered is the refusal by users to be continuously monitored” was found to be closely related to “security and privacy” items SP4 and SP6.

On the other hand, some experts commented on aspects of the existing categories but pointed out new visions, so they were incorporated as new items in the following rounds. Statements, such as “The use of work-related mHealth tools should be integrated into workday tasks” and “To facilitate the use of mHealth tools during the workday, they should be brief in nature” (“how should mHealth devices be” items H11 and H12, respectively), are examples of this.

#### Round 2

The second round consisted of 15 items, including 10 items not agreed upon in the previous round, 2 new items formulated from the answers to the previous round’s open-ended questions included in the section “how should mHealth devices be,” and 3 items included in the new “adherence” section.

After the survey was presented to experts, the categories “how should mHealth devices be” and “adherence” achieved consensus. However, the categories “occupational health” and “security and privacy” did not achieve the minimum 75% consensus; thus, they were included in the third round.

#### Round 3

The third round consisted of 5 items that did not reach a consensus in the previous round, including 3 items in the “occupational health” category and 2 items in the “security and privacy” category. Again, consensus was not achieved in this third round either. The results show disagreement given the wide dispersion of responses.

### Data Analysis

In Tables 2-6, we present all the items in the 3 rounds (translated into English) and the results. The tables also show whether agreement was achieved. Moreover, the tables show which option achieved the most remarkable consensus and which of the options received the most votes. Furthermore, the arithmetic means that characterize the central tendency and the SDs that measure the dispersion are indicated. The original survey in Spanish is available in Multimedia Appendix 2.

The results are discussed in greater depth in Multimedia Appendix 3. The statistical measures of mean, mode, maximum, and minimum for each item of all rounds are included in this appendix.

Our data revealed that the categories “procedure (applicability)” and “how should mHealth devices be” most easily reached an agreement. The “procedure (applicability)” category reached absolute consensus in the first round, and only 1 item in the “how should mHealth devices be” category had to be resubmitted a second time before reaching the established consensus threshold. In the second round, 2 new items were added based on the responses obtained in the open-ended questions and were consensual in the second round. The “adherence” category, incorporated into the questionnaire in round 2, was created after analysis of the opinions contributed by the experts to the open-ended questions in round 1, and its items achieved consensus the first time they were presented.

However, several items in the categories of “occupational health” and “safety and privacy” did not achieve consensus. In the “occupational health” category, 5 items reached consensus in round 1 and 2 more items reached consensus in round 2. After this second round, the experts continued to disagree on 3 items, and these items represented major points of disagreement. In the “security and privacy” category, 6 items reached consensus in round 1 and 2 more items reached consensus in round 2. After round 3, there were still strong disagreements for 2 items in the “security and privacy” category.

**Figure 3:**
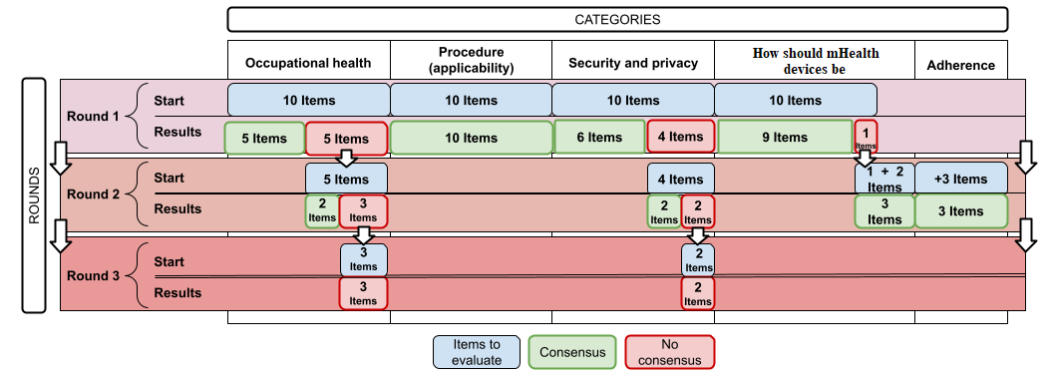
Graphical representation of the Delphi process. mHealth: mobile health.

Finally, after the 3 iterative rounds, the experts did not agree on 5 items. Analyses were carried out according to the field of knowledge to which the experts belonged, but there were no notable findings regarding these 5 items. For a better understanding, this complete process is graphically represented in Figure 3.

**Figure 4:**
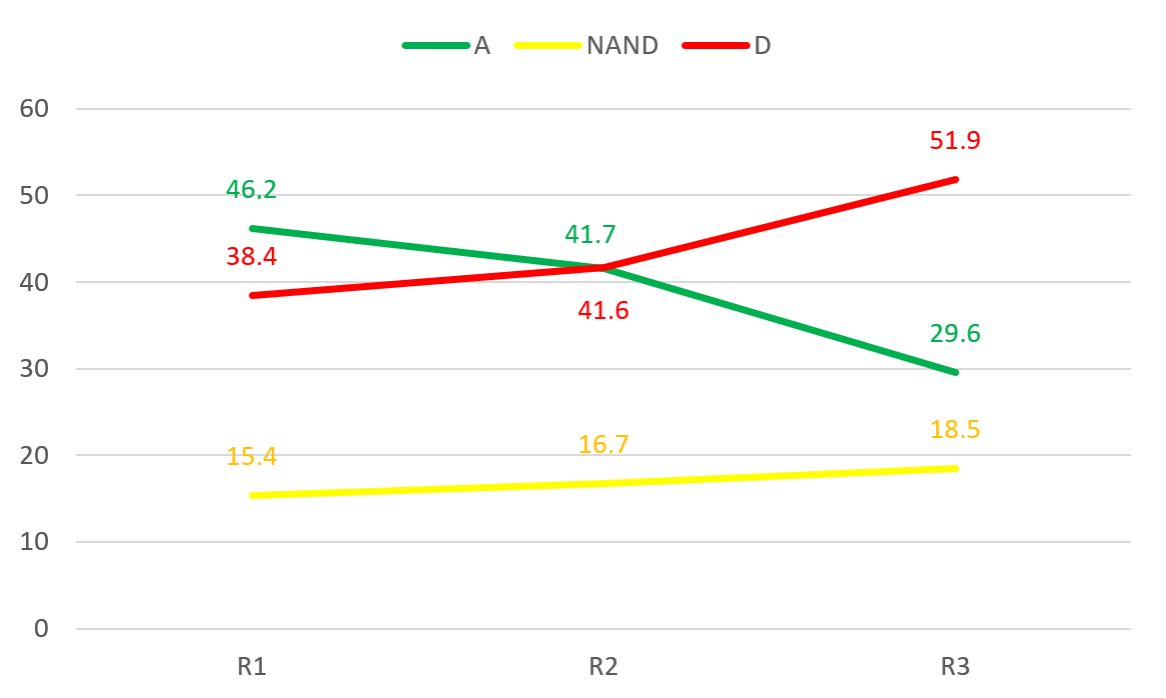
Iterations of item OH7 in the “occupational health” category in each round. A: agree; D: disagree; NAND: neither agree nor disagree; R: round.

**Figure 5:**
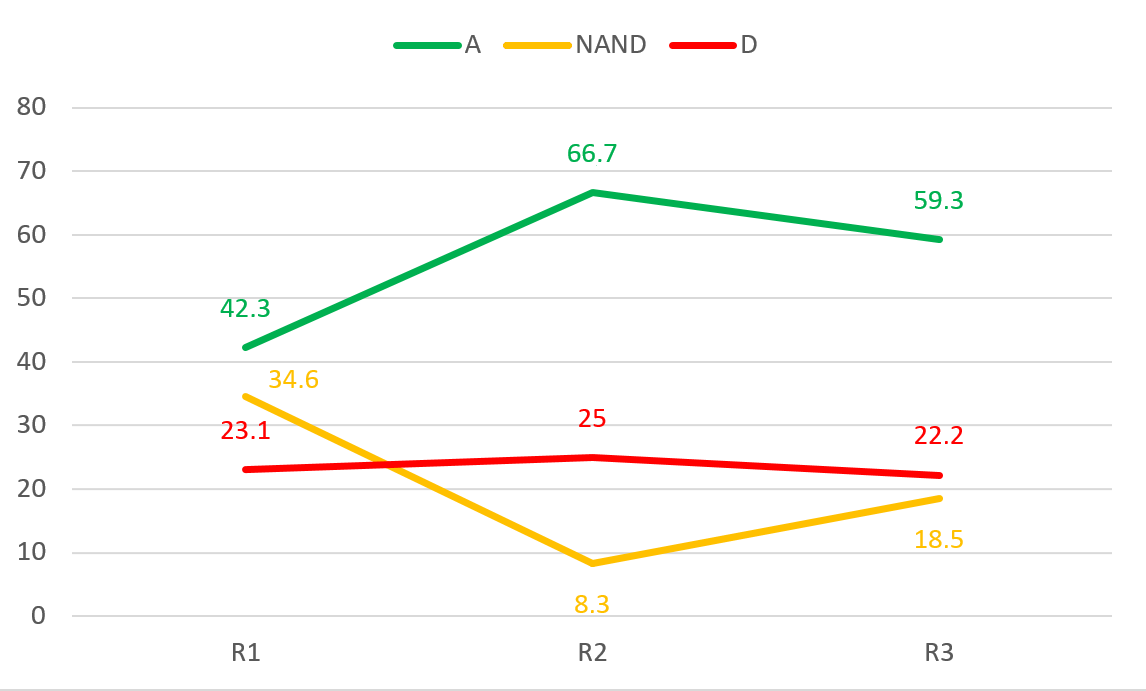
Iterations of item OH9 in the “occupational health” category in each round. A: agree; D: disagree; NAND: neither agree nor disagree; R: round.

**Figure 6:**
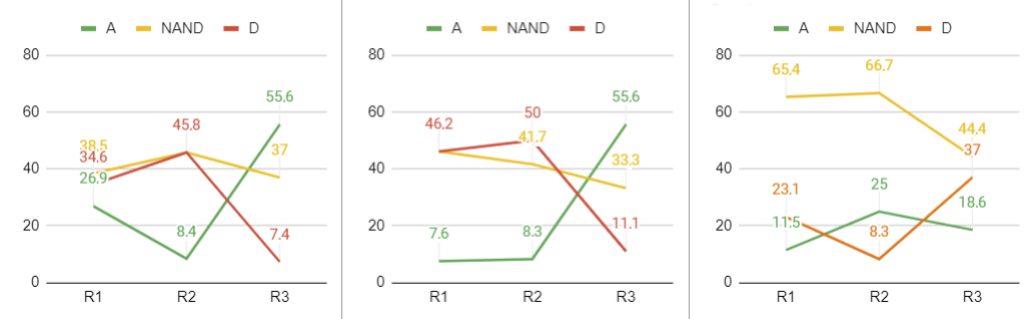
Iterations of item OH10 in the “occupational health” category, and items SP3 and SP4 in the “security and privacy” category in each round. A: agree; D: disagree; NAND: neither agree nor disagree; R: round.

Detailed results are provided for nonconsensus items in Figures 4-6. For item OH7 in the “occupational health” category, there seemed to be a trend toward dissensus. Thus, more iterations might result in dissensus, and “neither agree nor disagree” responses might grow with iterations. Therefore, it cannot be concluded that a consensus will be reached.

For item OH9 in the “occupational health” category, there was no clear trend, but the majority agreed with the item. With more iterations, the 75% agreement required for consensus might not be reached.

For item OH10 in the “occupational health” category, and items SP3 and SP4 in the “security and privacy” category, no clear trends were identified, mainly because a high percentage of respondents did not have a clear position between agreement and disagreement. Therefore, further iterations might not allow agreement to be reached.

There is no apparent reason for the lack of consensus beyond the one identified above for item OH9 in the “occupational health” category and item SP4 in the “security and privacy” category. It may be necessary to conduct a further study to identify the reasons for not reaching a consensus on these items.

**Table 2:** Items and results of the “occupational health” category (English version).

Occupational health (OH) category items	Results							
	Round 1			Round 2			Round 3	
	Consensus yes (≥75%) or no (A^a^, NAND^b^, or D^c^ %)	Mean (SD)	Consensus yes (≥75%) or no (A, NAND, or D %)		Mean (SD)	Consensus yes (≥75%) or no (A, NAND, or D %)		Mean (SD)
OH1: It is crucial for employees to have occupational health information available to them.	Yes (A=96.2%)	4.65 (0.85)	N/A^d^		N/A	N/A		N/A
OH2: It is necessary to evaluate the occupational health of professionals.	Yes (A=96.2%)	4.69 (0.84)	N/A		N/A	N/A		N/A
OH3: Professionals have the right to have their occupational health protected.	Yes (A=96.2%)	4.73 (0.84)	N/A		N/A	N/A		N/A
OH4: Detecting stress levels can help improve the well-being of workers.	Yes (A=96.2%)	4.65 (0.85)	N/A		N/A	N/A		N/A
OH5: Mobile health (mHealth) devices are more productive resources for conducting occupational risk prevention (ORP) assessments than conventional methods.	No (A=7.7%, NAND=42.3%, D=50.0%)	3.73 (1.00)	Yes (A=79.2%)		3,88 (0.54)	N/A		N/A
OH6: mHealth devices have transformed the way professionals evaluate aspects of ORP.	No (A=11.5%, NAND=30.8%, D=57.7%)	3.62 (1.02)	Yes (A=75.0%)		3.79 (0.78)	N/A		N/A
OH7: Organizations are aware of the importance of taking care of the occupational health of employees.	No (A=46.2%, NAND=15.4%, D=38.4%)	3.00 (1.26)	No (A=41.7%, NAND=16.7%, D=41.7%)		3.91 (1.12)	No (A=29.6%, NAND=18.5%, D=51.9%)		3.30 (0.99)
OH8: The fact that organizations evaluate the occupational health of their professionals gives them a sense of being taken into account.	Yes (A=84.7%)	4.19 (0.94)	N/A		N/A	N/A		N/A
OH9: The use of technology to evaluate occupational health and safety information is supported in organizations.	No (A=42.3%, NAND=34.6%, D=23.1%)	2.81 (1.06)	No (A=66.7%, NAND=8.3%, D=25.0%)		3.92 (0.93)	No (A=59.3%, NAND=18.5%, D=22.2%)		2.56 (0.93)
OH10: Currently, organizations take corrective actions when problems are detected in the occupational health of their workers.	No (A=26.9%, NAND=38.5%, D=34.6%)	3.12 (0.86)	No (A=8.3%, NAND=45.8%, D=45.8%)		3.95 (0.72)	No (A=55.6%, NAND=37.0%, D=7.4%)		3.59 (0.80)

^a^A: agree.

^b^NAND: neither agree nor disagree.

^c^D: disagree.

^d^N/A: not applicable.

**Table 3:** Items and results of the “procedure (applicability)” category (English version).

Procedure (applicability) (P) category items	Results									
	Round 1			Round 2			Round 3			
	Consensus yes (≥75%) or no (A^a^, NAND^b^, or D^c^ %)	Mean (SD)	Consensus yes (≥75%) or no (A, NAND, or D %)		Mean (SD)	Consensus yes (≥75%) or no (A, NAND, or D %)			Mean (SD)	
P1: Technical assistance should be provided to professionals during the monitoring period if necessary.	Yes (A=100%)	4.69 (0.47)	N/A^d^		N/A	N/A		N/A		
P2: Mobile health (mHealth) devices should be periodically checked to ensure proper functioning.	Yes (A=100%)	4.73 (0.45)	N/A		N/A	N/A		N/A		
P3: Professionals providing information about their occupational health should be included in the working day.	Yes (A=88.4%)	4.35 (0.80)	N/A		N/A	N/A		N/A		
P4: Professionals should receive an informative day where they are taught how to use mHealth devices for occupational health.	Yes (A=100%)	4.77 (0.43)	N/A		N/A	N/A		N/A		
P5: To encourage participation in the occupational health study, professionals should feel supported by their supervisors.	Yes (A=96.2%)	4.77 (0.51)	N/A		N/A	N/A		N/A		
P6: In the occupational health assessment, the adherence of workers can be increased by providing feedback about their participation.	Yes (A=92.3%)	4.73 (0.60)	N/A		N/A	N/A		N/A		
P7: The study of the occupational health of professionals should take into account the perspective of gender.	Yes (A=77.0%)	4.19 (1.06)	N/A		N/A	N/A		N/A		
P8: mHealth devices can be used to record variables that evaluate people’s occupational health.	Yes (A=92.3%)	4.62 (0.64)	N/A		N/A	N/A		N/A		
P9: Occupational stress can be detected by recording physiological variables and psychological questionnaires.	Yes (A=100%)	4.58 (0.50)	N/A		N/A	N/A		N/A		
P10: Thanks to mHealth devices, professionals will be able to visualize the results of their records and make changes in their work habits.	Yes (A=92.3%)	4.42 (0.64)	N/A		N/A	N/A		N/A		

^a^A: agree.

^b^NAND: neither agree nor disagree.

^c^D: disagree.

^d^N/A: not applicable.

**Table 4:** Items and results of the “security and privacy” category (English version).

Security and privacy (SP) category items	Results							
	Round 1			Round 2			Round 3	
	Consensus yes (≥75%) or no (A^a^, NAND^b^, or D^c^ %)	Mean (SD)	Consensus yes (≥75%) or no (A, NAND, or D %)		Mean (SD)	Consensus yes (≥75%) or no (A, NAND, or D %)		Mean (SD)
SP1: The data collected must comply with current regulations on security and privacy (Constitutional Act 3/2018, of 5 December on Personal Data Protection and Guarantee of Digital Rights).	Yes (A=100%)	4.85 (0.37)	N/A^d^		N/A	N/A		N/A
SP2: Organizations must ensure the privacy of their workers in the recording of their physiological and psychological indicators.	Yes (A=100%)	4.92 (0.27)	N/A		N/A	N/A		N/A
SP3: Recording personal information through these mobile apps is secure.	No (A=7.6%, NAND=46.2%, D=46.2%)	3.62 (1.06)	No (A=8.3%, NAND=41.7%, D=50.0%)		3.46 (0.88)	No (A=55.6%, NAND=33.3%, D=11.1%)		3.52 (0.80)
SP4: Professionals agree to be monitored to assess their occupational health.	No (A=11.5%, NAND=65.4%, D=23.1%)	3.15 (0.67)	No (A=25.0%, NAND=66.7%, D=8.3%)		2.83 (0.56)	No (A=18.5%, NAND=44.4%, D=37.0%)		2.63 (1.01)
SP5: Recording physiological information in an ongoing and outside workplace may be construed as an invasion of privacy.	No (A=11.5%, NAND=15.4%, D=73.1%)	3.85 (1.16)	Yes (A=91.7%)		3.88 (0.80)	N/A		N/A
SP6: Practitioners agree to wear the wearable device throughout the day, if necessary, for the recording period.	No (A=15.4%, NAND=61.5%, D=23.1%)	3.04 (0.72)	Yes (NAND=72.9%)		2.96 (0.46)	N/A		N/A
SP7: Data collected must be kept confidential and analyzed in aggregate to avoid the identification of participants.	Yes (A=80.8%)	4.35 (0.98)	N/A		N/A	N/A		N/A
SP8: Employees may prefer to answer questions about their work reality via a mobile app rather than express it to a person, as might be the case with conventional methods.	Yes (A=77.0%)	3.92 (0.74)	N/A		N/A	N/A		N/A
SP9: Workers may fear being identified when responding truthfully to questions about their work environment.	Yes (A=96.2%)	4.23 (0.51)	N/A		N/A	N/A		N/A
SP10: Workers may be reluctant to share information about their psychological state.	Yes (A=100%)	4.46 (0.51)	N/A		N/A	N/A		N/A

^a^A: agree.

^b^NAND: neither agree nor disagree.

^c^D: disagree.

^d^N/A: not applicable.

**Table 5:** Items and results of the “how should mHealth devices be” category (English version).

How should mHealth devices be (H) category items	Results							
	Round 1			Round 2			Round 3	
	Consensus yes (≥75%) or no (A^a^, NAND^b^, or D^c^ %)	Mean (SD)	Consensus yes (≥75%) or no (A, NAND, or D %)		Mean (SD)	Consensus yes (≥75%) or no (A, NAND, or D %)		Mean (SD)
H1: A mobile health (mHealth) device should be simple, intuitive, and easy to operate.	Yes (A=100%)	4.88 (0.33)	N/A^d^		N/A	N/A		N/A
H2: An instrument to measure occupational health should detect stressful situations and tasks.	Yes (A=92.3%)	4.54 (0.65)	N/A		N/A	N/A		N/A
H3: An app to measure occupational health should generate notifications when there are high-stress levels.	Yes (A=92.3%)	4.58 (0.64)	N/A		N/A	N/A		N/A
H4: The wearable device must be accurate in measuring physiological variables in any activity.	Yes (A=96.2%)	4.65 (0.56)	N/A		N/A	N/A		N/A
H5: The wearable device must be able to collect data, even if offline or without coverage.	Yes (A=92.3%)	4.54 (0.65)	N/A		N/A	N/A		N/A
H6: A wearable device should be comfortable to wear for long periods and not get in the way of getting the job done.	Yes (A=96.2%)	4.77 (0.51)	N/A		N/A	N/A		N/A
H7: Interventions to improve occupational health should be of a short duration (around 15 minutes).	No (A=19.2%, NAND=38.5%, D=42.3%)	3.38 (1.10)	Yes (A=79.2%)		3.92 (0.58)	N/A		N/A
H8: It is positive that mHealth devices are customizable according to the needs of professionals.	Yes (A=80.8%)	4.15 (0.97)	N/A		N/A	N/A		N/A
H9: Participants in an mHealth study should have access to the history of their activities and physiological signals to be aware of their health status.	Yes (A=77.0%)	4.19 (0.90)	N/A		N/A	N/A		N/A
H10: The battery of wearable devices that perform occupational health measurements should have a long life.	Yes (A=88.5%)	4.54 (0.71)	N/A		N/A	N/A		N/A
H11: The use of work-related mHealth tools should be integrated into workday tasks.	Not included in round 1	N/A	Yes (A=91.7%)		4.29 (0.81)	N/A		N/A
H12. To facilitate mHealth tools during the workday, they should be brief.	Not included in round 1	N/A	Yes (NAND=79.2%)		4.04 (0.81)	N/A		N/A

^a^A: agree.

^b^NAND: neither agree nor disagree.

^c^D: disagree.

^d^N/A: not applicable.

**Table 6:** Items and results of the “adherence” category (English version).

Adherence (Ad) category items	Results							
	Round 1			Round 2			Round 3	
	Consensus yes (≥75%) or no (A^a^, NAND^b^, or D^c^ %)	Mean (SD)	Consensus yes (≥75%) or no (A, NAND, or D %)		Mean (SD)	Consensus yes (≥75%) or no (A, NAND, or D %)		Mean (SD)
Ad1: It is crucial to include end-user feedback during the development of mobile health (mHealth) tools.	Not included in round 1	N/A^d^	Yes (A=100%)		4.71 (0.46)	N/A		N/A
Ad2: Adherence to mHealth tools will improve if practitioners perceive that their superiors support their use.	Not included in round 1	N/A	Yes (A=79.2%)		4.29 (0.81)	N/A		N/A
Ad3: Conducting training sessions on how to use mHealth tools can encourage their use.	Not included in round 1	N/A	Yes (A=95.8%)		4.50 (0.59)	N/A		N/A

^a^A: agree.

^b^NAND: neither agree nor disagree.

^c^D: disagree.

^d^N/A: not applicable.

## Discussion

### Principal Findings

The main findings of this study suggest that the use of mHealth tools in occupational health assessments allows for understanding and managing occupational health in organizations. Owing to the participation of the experts consulted, this study identified the main valuable characteristics that should be taken into account for organizations to use mHealth technologies appropriately with their employees. It is considered very necessary to assess the occupational health of professionals, considering that knowing the levels of stress can help improve the well-being of employees [31].

Making use of new technologies as an intrinsic approach for organizations to perform evaluations in the field of occupational risk prevention is appropriate. Therefore, data collection and analysis make it possible to identify and prevent risks related to the workplace [32]. The experts surveyed in our study were convinced that making occupational health evaluations available to the team will positively affect employees by increasing their feelings of being taken into account by the organization. In this sense, it should be noted that our experts believe that for professionals to participate in occupational health studies, it is necessary that they feel supported by their supervisors. Besides, Chin et al [33] in fact stated that social influences from colleagues, employers, and health care professionals can exert a strong effect on the intention to use a personal health record app in a workplace setting.

However, not everyone agreed on the support provided by organizations to use technology to assess occupational health and safety information, according to item OH9 in the “occupational health” category. In this sense, some defended the use of technology. This finding is consistent with the finding by Jimenez and Brezenger [34] who noted that lack of management support was considered a barrier to using these tools or a facilitator whenever there was strong management support. Moreover, other experts did not consider that the use of mobile apps to record personal information on the job was safe. They feared that employees might be reluctant to share information about their psychological state or that workers could be identified when answering specific questions. This is justified by the dissent in item SP4 (Professionals agree to be monitored to assess their occupational health) in the “security and privacy” category. Similarly, according to some of them, recording physiological information continuously and outside of the work environment makes it difficult for professionals to accept being evaluated and monitored. In the open-ended questions, some participants commented “Managing the fear of staff control by the organization is an element to overcome” and “One of the transversal difficulties that can be encountered is the refusal of users to be continuously monitored.” In contrast, there are experiences in which our participants themselves revealed that real-time monitoring is beneficial because it would help team management by allowing immediate intervention as soon as any difficulty is detected, without the need to wait until the end of the work shift [35]. Other authors have suggested that employers also benefit because working conditions and employee health can be tracked in order to implement health measures tailored to the workforce, thus improving occupational safety by preventing accidents [36].

On the other hand, the results of this study showed that, in order to achieve user satisfaction and acceptance of technology, devices must be simple, intuitive, and easy to use, in addition to meeting training requirements and the perception of health professionals of the quality of these applications [37].

Similarly, according to the consensus agreement on item P4 in the “procedure (applicability)” category, the participants considered it essential that professionals receive training. This would facilitate the use of the devices and, at the same time, improve adherence (item Ad3 in the “adherence” category). Another shared opinion is that receiving feedback on the records made can be helpful. Regarding ethical and privacy aspects, all experts agreed that occupational health must always be protected and that current regulations must be respected. In this regard, the vast majority of experts considered it essential to ensure the confidentiality of the collected data and to inform employees that the collected information will preserve the privacy of the workers and the integrity of the data. To achieve this objective, some authors advocate applying data aggregation techniques to guarantee privacy [38].

Finally, the experts came to a consensus that the most appropriate moment to assess psychosocial risks and occupational health is during working hours. We suppose this is related to achieving more adherence, and it is feared that they would not participate properly. However, there are discrepancies in considering that occupational health should be assessed with such devices. Some experts questioned the current efforts of organizations to take care of the occupational health of their employees. In 2010, the World Health Organization referred to a “healthy workplace” as one in which workers collaborate to implement a continuous improvement process to protect and promote the health, safety, and well-being of all employees [39]. However, there seemed to be no agreement on how organizations apply corrective actions after detecting problems in the occupational health of their workers. In the literature, we found that taking action as soon as problems are detected is imperative, as there is evidence of higher rates of absenteeism and conflicts in organizations that do not manage stress levels and other occupational health problems. Similarly, burnout syndrome gradually increases as productivity and individual performance decrease [40].

### Limitations and Strengths

We mainly identified limitations related to the application of the Delphi method. First, the creation of the expert panel was limited to the environment known to the study’s researchers. However, it did not prevent the study from complying with established recommendations on the size of the Delphi panel and the selection of experts. Those people who were considered suitable to respond to the questions raised in the survey were identified (once their professional backgrounds were known). Second, the period in which the iterative rounds were carried out may have had an impact. The rounds were carried out during the summer months, which coincided with the experts’ vacation periods, and the periods between rounds were longer, which could have affected the answers they gave to items presented to them repeatedly. Third, limiting the number of iterations to a maximum of 3 to reach consensus can be considered a limitation. This may have prevented consensus from being reached. According to the results of the nonconsensus items, there was no apparent convergence after the 3 iterations, which suggests that consensus will not be reached efficiently.

Our study has some strengths. To our knowledge, there are hardly any studies in which experts have been asked about the most important characteristics of an mHealth tool to be useful for assessing the health of members of organizations, while at the same time being accepted by potential users to improve their well-being at work. Given the professional background, theoretical and practical knowledge, and experience of the experts invited to our survey, they were considered to have the capacity to make informed judgements about the use of digital health devices to assess the stress levels and burnout experienced by health care professionals. The importance of our study lies in the use of an mHealth tool to assess stress and other psychosocial risks over other kinds of tools, such as surveys, and provide immediate feedback about the levels of these measures to health care professionals. This feedback could help to regulate negative states and identify resources to cope with the situation.

### Conclusions

As a result of the findings, organizations should be aware of the importance of assessing the occupational health of their employees. Failure to do so can pose a threat to the health of workers. In addition, extreme working conditions, such as those caused by the COVID-19 pandemic in the health sector, pose a huge risk to occupational health. This makes it urgently necessary to know and control the psychosocial risks to which workers are exposed.

The application of mHealth systems to occupational health can be a major step forward in evaluation. Our work has focused on taking these aspects into account when implementing occupational health tools. We conclude that mHealth apps should primarily contain systems for evaluating the parameters for determining occupational health, including indicators on occupational health and user performance. There should be information and training on occupational health, information on the use of the mHealth app itself, and information on compliance with applicable data protection and privacy legislation. There should also be a user support service. Other content features identified by experts include the identification of stressful situations and the detection and notification of tasks. In relation to the use of wearable devices, accuracy, comfort, and durability were identified as important aspects.

Moreover, the experts were concerned about the right to occupational health protection and the protection and privacy of data and users. They also identified the usability and reliability of the app’s operation, including stress detection, as important concerns, always taking into account the time of data collection, which should be done within the working day, but without being a distraction or an added burden to daily tasks. The experts noted that the support of supervisors is important for the implementation of such apps and for the commitment and adherence of workers to use these tools. They also noted that including end users in the design of these tools is key to ensuring that they meet utility and usability criteria.

mHealth tools provide support and resources to organizations for managing occupational risks. The use of mHealth tools helps to identify hazards and risks in the workplace and facilitates the risk assessment process, and these tools are easy to use. Accessing and interacting with the apps are not barriers as they are installed on users’ own mobile phones, tablets, etc, making it easy to connect with and reach a wide range of audiences. mHealth opens the door to the evaluation of employees’ occupational health. In the past, mHealth tools have mainly been used to propose intervention programs, but nowadays, it is possible to use them in the preliminary phase of the psychosocial risk assessment of occupational health.

## Appendix

Multimedia Appendix 1 Definitions of words used in the survey.

Multimedia Appendix 2 Original survey items in Spanish and results: degree of consensus, arithmetic mean, and standard deviation.

Multimedia Appendix 3 Results of each round (Spanish version): mean, mode, maximum, and minimum of each item.

## Abbreviations

mHealth: mobile health

## Data Availability

Our data set has been deposited in publicly accessible repositories. It is accessible via Zenodo [41].
