# Supplementary material for: Mobile Health Requirements for the Occupational Health Assessment of Health Care Professionals: Delphi Study
Source: JMIR Form Res. 2023 May 31;7:e40327. doi: 10.2196/40327 (PMC10267780; doi:10.2196/40327)
Supplement: Multimedia Appendix 1 [file formative_v7i1e40327_app1.docx]

**Multimedia Appendix 1.** Definitions of words used in the survey.

- mHealth: the practice of medicine and public health supported by mobile devices, such as cell phones, monitoring devices, personal digital assistants, and other wireless devices.

- Occupational Health: the activity aims to maintain the best level of physical, psychological, and social well-being of the working population in all professions and prevent any damage due to their work conditions.

- Wearable: devices that are integrated into clothing or accessories such as watches and bracelets.
