# Supplementary material for: Mobile Health Requirements for the Occupational Health Assessment of Health Care Professionals: Delphi Study
Source: JMIR Form Res. 2023 May 31;7:e40327. doi: 10.2196/40327 (PMC10267780; doi:10.2196/40327)
Supplement: Multimedia Appendix 2 [file formative_v7i1e40327_app2.docx]

**Multimedia Appendix 2.** Original survey items in Spanish and results: degree of consensus, arithmetic mean, and standard deviation.

| **ITEMS** | **RESULTS** | | | | | |
| --- | --- | --- | --- | --- | --- | --- |
|  | **Round 1** | | **Round 2** | | **Round 3** | |
|  | **Consensus YES ( ≥75%)/ No (A, NAND, D)** | **Mean (SD*)** | **Consensus ( ≥75%)/ No (A, NAND, D)** | **Mean (SD*)** | **Consensus (≥75%)/ No (A, NAND, D)** | **Mean (SD*)** |
| **Occupational health - OH** |  |  |  |  |  |  |
| OH1. Es importante que los empleados tengan a su disposición información sobre salud laboral | **Yes, 96,2% (A)** | 4,65 (0,85) |  |  |  |  |
| OH2. Es necesario que se evalúe la salud laboral de los profesionales. | **Yes, 96,2% (A)** | 4,69 (0,84) |  |  |  |  |
| OH3. Los profesionales tienen derecho a que su salud laboral sea protegida. | **Yes, 96,2% (A)** | 4,73 (0,84) |  |  |  |  |
| OH4. Detectar los niveles de estrés puede ayudar a mejorar el bienestar de los trabajadores. | **Yes, 96,2% (A)** | 4,65 (0,85) |  |  |  |  |
| OH5. Los dispositivos de mHealth son recursos más productivos para hacer evaluaciones sobre Prevención de Riesgos Laborales (PRL) que los métodos convencionales. | No  (A= 7,7%  NAND= 42,3%  D=50%) | 3,73 (1) | **Yes, 79,2% (A)** | 3,88  (0,54) |  |  |
| OH6. Los dispositivos de mHealth han transformado la manera de evaluar aspectos de PRL en los profesionales. | No  (A= 11,5%  NAND= 30,8%  D=57,7%) | 3,62 (1,02) | **Yes, 75,0% (A)** | 3,79  (0,78) |  |  |
| OH7. Las organizaciones están concienciadas de la importancia de cuidar la salud laboral de los empleados. | No  (A= 46,2%  NAND= 15,4%  D=38,4%) | 3,00 (1,26) | No  (A= 41,7%  NAND= 16,7%  D=41,7%) | 3,91  (1,12) | No  (A= 29,63%  NAND= 18,5%  D=51,9%) | 3,30  (0,99) |
| OH8. Que las organizaciones evalúen la salud laboral de sus profesionales les da a estos sensación de ser tenidos en cuenta. | **Yes, 84,7% (A)** | 4,19 (0,94) |  |  |  |  |
| OH9. En las organizaciones se apoya el uso de la tecnología para evaluar información de Prevención de Riesgos Laborales. | No  (A= 42,3%  NAND= 34,6%  D=23,1%) | 2,81 (1,06) | No  (A= 66,7%  NAND= 8,3%  D=25%) | 3,92  (0,93) | No  (A= 59,3%  NAND= 18,5%  D=22,2%) | 2,56  (0,93) |
| OH10. Actualmente, las organizaciones toman acciones correctivas cuando se detectan problemas en la salud laboral de sus trabajadores. | No  (A= 26,9%  NAND= 38,5%  D=34,6%) | 3,12 (0,86) | No  (A= 8,3%  NAND= 45,8%  D=45,8%) | 3,95  (0,72) | No  (A= 55,6%  NAND= 37%  D=7,4%) | 3,59  (0,80) |
| **Procedure (applicability)-P** |  |  |  |  |  |  |
| P1. Se debe facilitar ayuda técnica a los profesionales durante el periodo de monitorización si la necesitan. | **Yes, 100,0% (A)** | 4,69 (0,47) |  |  |  |  |
| P2. Los dispositivos de mHealth deben ser periódicamente revisados para asegurar su correcto funcionamiento. | **Yes, 100,0% (A)** | 4,73 (0,45) |  |  |  |  |
| P3. Que los profesionales aporten información acerca de su salud laboral debe estar contemplado dentro de la jornada del trabajo. | **Yes, 88,4% (A)** | 4,35 (0,8) |  |  |  |  |
| P4. Los profesionales deben recibir una jornada informativa donde se les enseñe a usar los dispositivos mHealth de salud laboral. | **Yes, 100,0% (A)** | 4,77 (0,43) |  |  |  |  |
| P5. Para favorecer la participación en el estudio de salud laboral, los profesionales deben sentirse apoyados por sus supervisores. | **Yes, 96,2% (A)** | 4,77 (0,51) |  |  |  |  |
| P6. En la evaluación de la salud laboral, se puede aumentar la adherencia de los trabajadores aportando feedback acerca de su participación. | **Yes, 92,3% (A)** | 4,73 (0,60) |  |  |  |  |
| P7. El estudio sobre la salud laboral de los profesionales debe tener en cuenta la perspectiva de género. | **Yes, 77,0% (A)** | 4,19 (1,06) |  |  |  |  |
| P8. Mediante dispositivos mHealth es posible registrar variables que evalúan la salud laboral de las personas. | **Yes, 92,3% (A)** | 4,62 (0,64) |  |  |  |  |
| P9. El estrés laboral se puede detectar a través del registro de variables fisiológicas y cuestionarios psicológicos. | **Yes, 100,0% (A)** | 4,58 (0,50) |  |  |  |  |
| P10. Gracias a dispositivos mHealth los profesionales podrán visualizar los resultados de sus registros y llevar a cabo cambios en sus hábitos laborales. | **Yes, 92,3% (A)** | 4,42 (0,64) |  |  |  |  |
| **Security and privacy - SP** |  |  |  |  |  |  |
| SP1. Los datos recogidos deben cumplir con la normativa vigente sobre seguridad y privacidad (Ley Orgánica 3/2018, de 5 de diciembre, de Protección de Datos Personales y garantía de los derechos digitales). | **Yes, 100,0% (A)** | 4,85  (0,37) |  |  |  |  |
| SP2. Las organizaciones deben asegurar la privacidad de sus trabajadores en el registro de sus indicadores fisiológicos y psicológicos. | **Yes, 100,0% (A)** | 4,92  (0,27) |  |  |  |  |
| SP3. Registrar información personal a través de estas aplicaciones móviles es seguro. | No  (A= 7,6%  NAND= 46,2%  D=46,2%) | 3,62  (1,06) | No  (A= 8,3%  NAND= 41,7%  D=50%) | 3,46  (0,88) | No  (A= 55,6%  NAND= 33,3%  D=11,1%) | 3,52  (0,80) |
| SP4. Los profesionales están de acuerdo en ser monitorizados para evaluar su salud laboral. | No  (A= 11,5%  NAND= 65,4%  D=23,1%) | 3,15  (0,67) | No  (A= 25%  NAND= 66,7%  D=8,3%) | 2,83  (0,56) | No  (A= 18,5%  NAND= 44,4%  D=37%) | 2,63  (1,01) |
| SP5. El registro de información fisiológica de manera continuada y fuera del entorno laboral puede interpretarse como una invasión de la intimidad. | No  (A= 11,5%  NAND= 15,4%  D=73,1%) | 3,85  (1,16) | **Yes, 91,7% (A)** | 3,88  (0,80) |  |  |
| SP6. Los profesionales están de acuerdo en llevar puesto el dispositivo wearable durante todo el día si es necesario, mientras dure el periodo de registro. | No  (A= 15,4%  NAND= 61,5%  D=23,1%) | 3,04  (0,72) | **Yes, 72,9% (NAND)** | 2,96  (0,46) |  |  |
| SP7. Los datos recogidos tienen que ser confidenciales y analizarse en conjunto para evitar la identificación de los participantes. | **Yes, 80,8% (A)** | 4,35  (0,98) |  |  |  |  |
| SP8. Los empleados pueden preferir responder cuestiones sobre su realidad laboral mediante una app móvil que expresarlo a una persona, como podría ocurrir en los métodos convencionales. | **Yes, 77,0% (A)** | 3,92  (0,74) |  |  |  |  |
| SP9. Los trabajadores pueden temer ser identificados al responder con sinceridad a cuestiones sobre su entorno laboral. | **Yes, 96,2% (A)** | 4,23  (0,51) |  |  |  |  |
| SP10. Los trabajadores pueden ser reticentes a compartir información sobre su estado psicológico. | **Yes, 100,0% (A)** | 4,46  (0,51) |  |  |  |  |
| **What mHealth devices should look like -W** |  |  |  |  |  |  |
| W1. Un dispositivo mHealth debe ser sencillo, intuitivo y fácil de manejar. | **Yes, 100,0% (A)** | 4,88  (0,33) |  |  |  |  |
| W2. Un instrumento para medir la salud laboral debe detectar las situaciones y tareas estresantes. | **Yes, 92,3% (A)** | 4,54  (0,65) |  |  |  |  |
| W3. Una aplicación para medir la salud laboral debe generar notificaciones ante elevados niveles de estrés. | **Yes, 92,3%** **(A)** | 4,58  (0,64) |  |  |  |  |
| W4. El dispositivo wearable debe ser preciso en la medición constante de las variables fisiológicas ante cualquier actividad. | **Yes, 96,2% (A)** | 4,65  (0,56) |  |  |  |  |
| W5. El dispositivo wearable debe poder recoger datos, aunque esté offline o no tenga cobertura. | **Yes, 92,3% (A)** | 4,54  (0,65) |  |  |  |  |
| W6. Un dispositivo wearable debe ser cómodo de llevar durante largos periodos de tiempo y no molestar para hacer el trabajo. | **Yes, 96,2% (A)** | 4,77  (0,51) |  |  |  |  |
| W7. Las intervenciones para mejorar la salud laboral deben ser de corta duración (alrededor de 15 minutos). | No  (A= 19,2%  NAND= 38,5%  D=42,3%) | 3,38  (1,10) | **Yes, 79,2% (A)** | 3,92  (0,58) |  |  |
| W8. Es positivo que los dispositivos mHealth sean personalizables según las necesidades de los profesionales. | **Yes, 80,8% (A)** | 4,15  (0,97) |  |  |  |  |
| W9. Los participantes en un estudio sobre mHealth deben tener acceso al historial de sus actividades y de sus señales fisiológicas para ser conscientes de su estado de salud. | **Yes, 77,0% (A)** | 4,19  (0,90) |  |  |  |  |
| W10. La batería de los dispositivos wearable que realizan mediciones de salud laboral debe ser de larga duración. | **Yes, 88,5% (A)** | 4,54  (0,71) |  |  |  |  |
| W11. El uso de las herramientas de mHealth relacionadas con el trabajo deben integrarse entre las tareas de la jornada laboral. | Not included in round 1 |  | **Yes, 91,7% (A)** | 4,29 (0,81) |  |  |
| W12. Para facilitar el uso de las herramientas m-Health durante la jornada laboral, estas deben tener un carácter breve. | Not included in round 1 |  | **Yes, 79,2% (NAND)** | 4,04  (0,81) |  |  |
| **Adherence -Ad** |  |  |  |  |  |  |
| Ad1. Es importante incluir la opinión de los usuarios finales durante la elaboración de las herramientas de mHealth. | Not included in round 1 |  | **Yes, 100,0% (A)** | 4,71  (0,46) |  |  |
| Ad2. La adherencia a las herramientas de mHealth mejorará si los profesionales perciben que sus superiores apoyan el uso de las mismas. | Not included in round 1 |  | **Yes, 79,2% (A)** | 4,29  (0,81) |  |  |
| Ad3. Realizar sesiones de formación acerca de cómo usar las herramientas de mHealth puede fomentar que se haga uso de ellas. | Not included in round 1 |  | **Yes, 95,8% (A)** | 4,50  (0,59) |  |  |
