# Supplementary material for: Mobile Health Requirements for the Occupational Health Assessment of Health Care Professionals: Delphi Study
Source: JMIR Form Res. 2023 May 31;7:e40327. doi: 10.2196/40327 (PMC10267780; doi:10.2196/40327)
Supplement: Multimedia Appendix 3 [file formative_v7i1e40327_app3.docx]

**Multimedia Appendix 3.** Results of each round (Spanish version): mean, mode, maximum, and minimum of each item.

| **ITEMS** | **RESULTS** | | | | | | | | | | | |
| --- | --- | --- | --- | --- | --- | --- | --- | --- | --- | --- | --- | --- |
|  | **Round 1** | | | | **Round 2** | | | | **Round 3** | | | |
|  | **Median** | **Mode** | **Maximum** | **Minimum** | **Median** | **Mode** | **Maximum** | **Minimum** | **Median** | **Mode** | **Maximum** | **Minimum** |
| **Salud laboral - SL** |  |  |  |  |  |  |  |  |  |  |  |  |
| SL1. Es importante que los empleados tengan a su disposición información sobre salud laboral | 5 | 5 | 5 | 1 |  |  |  |  |  |  |  |  |
| SL2. Es necesario que se evalúe la salud laboral de los profesionales. | 5 | 5 | 5 | 1 |  |  |  |  |  |  |  |  |
| SL3. Los profesionales tienen derecho a que su salud laboral sea protegida. | 5 | 5 | 5 | 1 |  |  |  |  |  |  |  |  |
| SL4. Detectar los niveles de estrés puede ayudar a mejorar el bienestar de los trabajadores. | 5 | 5 | 5 | 1 |  |  |  |  |  |  |  |  |
| SL5. Los dispositivos de mHealth son recursos más productivos para hacer evaluaciones sobre Prevención de Riesgos Laborales (PRL) que los métodos convencionales. | 3,5 | 3 | 5 | 2 | 4 | 4 | 5 | 3 |  |  |  |  |
| SL6. Los dispositivos de mHealth han transformado la manera de evaluar aspectos de PRL en los profesionales. | 4 | 4 | 5 | 1 | 4 | 4 | 5 | 2 |  |  |  |  |
| SL7. Las organizaciones están concienciadas de la importancia de cuidar la salud laboral de los empleados. | 3 | 2 | 5 | 1 | 3 | 2 | 5 | 2 | 4 | 4 | 5 | 2 |
| SL8. Que las organizaciones evalúen la salud laboral de sus profesionales les da a estos sensación de ser tenidos en cuenta. | 4 | 5 | 5 | 1 |  |  |  |  |  |  |  |  |
| SL9. En las organizaciones se apoya el uso de la tecnología para evaluar información de Prevención de Riesgos Laborales. | 3 | 3 | 5 | 1 | 2 | 2 | 4 | 1 | 2 | 2 | 4 | 1 |
| SL10. Actualmente, las organizaciones toman acciones correctivas cuando se detectan problemas en la salud laboral de sus trabajadores. | 3 | 3 | 5 | 2 | 3 | 3 | 5 | 2 | 4 | 4 | 5 | 2 |
| **Procedimiento (aplicabilidad) - P** |  |  |  |  |  |  |  |  |  |  |  |  |
| P1. Se debe facilitar ayuda técnica a los profesionales durante el periodo de monitorización si la necesitan. | 5 | 5 | 5 | 4 |  |  |  |  |  |  |  |  |
| P2. Los dispositivos de mHealth deben ser periódicamente revisados para asegurar su correcto funcionamiento. | 5 | 5 | 5 | 4 |  |  |  |  |  |  |  |  |
| P3. Que los profesionales aporten información acerca de su salud laboral debe estar contemplado dentro de la jornada del trabajo. | 4,5 | 5 | 5 | 2 |  |  |  |  |  |  |  |  |
| P4. Los profesionales deben recibir una jornada informativa donde se les enseñe a usar los dispositivos mHealth de salud laboral. | 5 | 5 | 5 | 4 |  |  |  |  |  |  |  |  |
| P5. Para favorecer la participación en el estudio de salud laboral, los profesionales deben sentirse apoyados por sus supervisores. | 5 | 5 | 5 | 3 |  |  |  |  |  |  |  |  |
| P6. En la evaluación de la salud laboral, se puede aumentar la adherencia de los trabajadores aportando feedback acerca de su participación. | 5 | 5 | 5 | 3 |  |  |  |  |  |  |  |  |
| P7. El estudio sobre la salud laboral de los profesionales debe tener en cuenta la perspectiva de género. | 5 | 5 | 5 | 2 |  |  |  |  |  |  |  |  |
| P8. Mediante dispositivos mHealth es posible registrar variables que evalúan la salud laboral de las personas. | 5 | 5 | 5 | 3 |  |  |  |  |  |  |  |  |
| P9. El estrés laboral se puede detectar a través del registro de variables fisiológicas y cuestionarios psicológicos. | 5 | 5 | 5 | 4 |  |  |  |  |  |  |  |  |
| P10. Gracias a dispositivos mHealth los profesionales podrán visualizar los resultados de sus registros y llevar a cabo cambios en sus hábitos laborales. | 4,5 | 5 | 5 | 3 |  |  |  |  |  |  |  |  |
| **Seguridad y privacidad -SP** |  |  |  |  |  |  |  |  |  |  |  |  |
| SP1. Los datos recogidos deben cumplir con la normativa vigente sobre seguridad y privacidad (Ley Orgánica 3/2018, de 5 de diciembre, de Protección de Datos Personales y garantía de los derechos digitales). | 5 | 5 | 5 | 4 |  |  |  |  |  |  |  |  |
| SP2. Las organizaciones deben asegurar la privacidad de sus trabajadores en el registro de sus indicadores fisiológicos y psicológicos. | 5 | 5 | 5 | 4 |  |  |  |  |  |  |  |  |
| SP3. Registrar información personal a través de estas aplicaciones móviles es seguro. | 3 | 3 | 5 | 1 | 3,5 | 3 | 5 | 1 | 4 | 4 | 5 | 2 |
| SP4. Los profesionales están de acuerdo en ser monitorizados para evaluar su salud laboral. | 3 | 3 | 5 | 2 | 3 | 3 | 4 | 2 | 3 | 3 | 4 | 1 |
| SP5. El registro de información fisiológica de manera continuada y fuera del entorno laboral puede interpretarse como una invasión de la intimidad. | 4 | 4 | 5 | 1 | 4 | 4 | 5 | 1 |  |  |  |  |
| SP6. Los profesionales están de acuerdo en llevar puesto el dispositivo wearable durante todo el día si es necesario, mientras dure el periodo de registro. | 3 | 3 | 4 | 1 | 3 | 3 | 4 | 2 |  |  |  |  |
| SP7. Los datos recogidos tienen que ser confidenciales y analizarse en conjunto para evitar la identificación de los participantes. | 5 | 5 | 5 | 2 |  |  |  |  |  |  |  |  |
| SP8. Los empleados pueden preferir responder cuestiones sobre su realidad laboral mediante una app móvil que expresarlo a una persona, como podría ocurrir en los métodos convencionales. | 4 | 4 | 5 | 2 |  |  |  |  |  |  |  |  |
| SP9. Los trabajadores pueden temer ser identificados al responder con sinceridad a cuestiones sobre su entorno laboral. | 4 | 4 | 5 | 3 |  |  |  |  |  |  |  |  |
| SP10. Los trabajadores pueden ser reticentes a compartir información sobre su estado psicológico. | 4 | 4 | 5 | 4 |  |  |  |  |  |  |  |  |
| **Cómo deben ser los dispositivos mHealth - CD** |  |  |  |  |  |  |  |  |  |  |  |  |
| CD1. Un dispositivo mHealth debe ser sencillo, intuitivo y fácil de manejar. | 5 | 5 | 5 | 4 |  |  |  |  |  |  |  |  |
| CD2. Un instrumento para medir la salud laboral debe detectar las situaciones y tareas estresantes. | 5 | 5 | 5 | 3 |  |  |  |  |  |  |  |  |
| CD3. Una aplicación para medir la salud laboral debe generar notificaciones ante elevados niveles de estrés. | 5 | 5 | 5 | 3 |  |  |  |  |  |  |  |  |
| CD4. El dispositivo wearable debe ser preciso en la medición constante de las variables fisiológicas ante cualquier actividad. | 5 | 5 | 5 | 3 |  |  |  |  |  |  |  |  |
| CD5. El dispositivo wearable debe poder recoger datos, aunque esté offline o no tenga cobertura. | 5 | 5 | 5 | 3 |  |  |  |  |  |  |  |  |
| CD6. Un dispositivo wearable debe ser cómodo de llevar durante largos periodos de tiempo y no molestar para hacer el trabajo. | 5 | 5 | 5 | 3 |  |  |  |  |  |  |  |  |
| CD7. Las intervenciones para mejorar la salud laboral deben ser de corta duración (alrededor de 15 minutos). | 3 | 3 | 5 | 1 | 4 | 4 | 5 | 3 |  |  |  |  |
| CD8. Es positivo que los dispositivos mHealth sean personalizables según las necesidades de los profesionales. | 4 | 5 | 5 | 1 |  |  |  |  |  |  |  |  |
| CD9. Los participantes en un estudio sobre mHealth deben tener acceso al historial de sus actividades y de sus señales fisiológicas para ser conscientes de su estado de salud. | 4 | 5 | 5 | 2 |  |  |  |  |  |  |  |  |
| CD10. La batería de los dispositivos wearable que realizan mediciones de salud laboral debe ser de larga duración. | 5 | 5 | 5 | 3 |  |  |  |  |  |  |  |  |
| CD11. El uso de las herramientas de mHealth relacionadas con el trabajo deben integrarse entre las tareas de la jornada laboral. | Not included in round 1 |  |  |  | 4 | 5 | 5 | 2 |  |  |  |  |
| CD12. Para facilitar el uso de las herramientas m-Health durante la jornada laboral, estas deben tener un carácter breve. | Not included in round 1 |  |  |  | 4 | 4 | 5 | 2 |  |  |  |  |
| **Adherencia - Ad** |  |  |  |  |  |  |  |  |  |  |  |  |
| Ad1. Es importante incluir la opinión de los usuarios finales durante la elaboración de las herramientas de mHealth. | Not included in round 1 Not included in round 1 |  |  |  | 5 | 5 | 5 | 4 |  |  |  |  |
| Ad2. La adherencia a las herramientas de mHealth mejorará si los profesionales perciben que sus superiores apoyan el uso de las mismas. | Not included in round 1 |  |  |  | 4,5 | 5 | 5 | 3 |  |  |  |  |
| Ad3. Realizar sesiones de formación acerca de cómo usar las herramientas de mHealth puede fomentar que se haga uso de ellas. | Not included in round 1 |  |  |  | 5 | 5 | 5 | 3 |  |  |  |  |
